# Quality of reporting in oncology phase II trials: A 5-year assessment through systematic review

**DOI:** 10.1371/journal.pone.0185536

**Published:** 2017-12-07

**Authors:** Julien Langrand-Escure, Romain Rivoirard, Mathieu Oriol, Fabien Tinquaut, Chloé Rancoule, Frank Chauvin, Nicolas Magné, Aurélie Bourmaud

**Affiliations:** 1 Centre Hygée, Public Health Department, Lucien Neuwirth Cancer Institut, Saint Priest en Jarez, France; 2 Radiotherapy Department, Lucien Neuwirth Cancer Institut, Saint Priest en Jarez, France; 3 Oncology Department, Lucien Neuwirth Cancer Institut, Saint Priest en Jarez, France; 4 INSERM 1408 CIC-EC, Saint Etienne, France; 5 EA HEalth Services Performance Research HESPER 7425, Lyon 1 University, Lyon, France; Georgia Regents University, UNITED STATES

## Abstract

**Background:**

Phase II clinical trials are a cornerstone of the development in experimental treatments They work as a "filter" for phase III trials confirmation. Surprisingly the attrition ratio in Phase III trials in oncology is significantly higher than in any other medical specialty. This suggests phase II trials in oncology fail to achieve their goal.

*Objective* The present study aims at estimating the quality of reporting in published oncology phase II clinical trials.

**Data sources:**

A literature review was conducted among all phase II and phase II/III clinical trials published during a 5-year period (2010–2015).

**Study eligibility criteria:**

All articles electronically published by three randomly-selected oncology journals with Impact-Factors>4 were included: Journal of Clinical Oncology, Annals of Oncology and British Journal of Cancer.

**Intervention:**

Quality of reporting was assessed using the Key Methodological Score.

**Results:**

557 articles were included. 315 trials were single-arm studies (56.6%), 193 (34.6%) were randomized and 49 (8.8%) were non-randomized multiple-arm studies. The Methodological Score was equal to 0 (lowest level), 1, 2, 3 (highest level) respectively for 22 (3.9%), 119 (21.4%), 270 (48.5%) and 146 (26.2%) articles. The primary end point is almost systematically reported (90.5%), while sample size calculation is missing in 66% of the articles. 3 variables were independently associated with reporting of a high standard: presence of statistical design (p-value <0.001), multicenter trial (p-value = 0.012), per-protocol analysis (p-value <0.001).

**Limitations:**

*S*creening was mainly performed by a sole author. The Key Methodological Score was based on only 3 items, making grey zones difficult to translate.

**Conclusions & implications of key findings:**

This literature review highlights the existence of gaps concerning the quality of reporting. It therefore raised the question of the suitability of the methodology as well as the quality of these trials, reporting being incomplete in the corresponding articles.

## Introduction

Phase II clinical trials are pivotal steps in the development of new drugs and/or new therapeutic strategies. In oncology particularly, the aim of phase II is to assess the efficacy and safeness of experimental treatments on a small sample of highly selected cancer patients, in order to determine an effective and safe enough dose, which could then be further administered to a broader population. Thus, phase II trials could be considered as a filter for experimental treatments, before phase III, defined as the largest scaled step and which is an obligatory stage before treatment registration.

Clinical research in oncology has evolved significantly in the last 20 years. While oncology was a discipline with a small number of experimental treatments in development, in the early 2000’s, the discovery of new systemic drugs, especially targeted therapies, and the technological progresses in radiation therapy have led to an explosion in the number of experimental treatments assessed in trials [1]. Lots of new drugs have been developed in oncology due to the urgent need to offer alternative and more efficient treatments to patients, but only a few of them have passed the stringent testing protocols of clinical research, especially during the phase II stage. Oncology is still the medical specialty with the highest attrition rate during the development of therapeutic treatment, all stages of development included. Particularly during phase III stage, oncology presents a mean attrition rate that is estimated to be 11.3% to 14% higher than the other specialties’ rates [2,3], leading to the hypothesis that oncology phase II trials do not successfully fulfill their filter role. Furthermore, since financial constraints are a fundamental issue in clinical research, there is a high rationale to try improve selection capacity of oncology phase II trials because of the important global cost linked to large phase III trials.

Several hypotheses may be raised to explain this peculiar phenomenon. One being that adequate methodology, specific to drug cancer research, may not be followed by investigators. This assumption may be supported by the fact that, at the methodological level, some discussion and general recommendations, essentially concerning the choice of the statistical design, have been published, [4–16], but up until today no consensus exists. The methodology used in oncology phase II trials responds to a non-systematic scheme. One way to initiate the investigation of this hypothesis is to explore the reporting of cancer phase II trials. Reporting is a key element to assessing the quality of trials, because it represents what is available for the readers. Concerning reporting, little data exist, and it is essentially made up of isolated methodological criteria and mainly reports the trial’s statistical design [17–23]. Until 2014, no global recommendation had been published for phase II trials, such as the CONSORT guidelines for randomized controlled trials [24–26]. Since January 2014, two global scores have been validated and published [27]. They are called the Overall Quality Score and the Key Methodological Score and are used to assess the quality of reporting in oncology phase II trials [27]. Investigating reporting with a validated tool would be the first step to a broader assessment of the methodological quality in phase II trials.

The aim of the present study was to describe and assess the reporting quality of oncology phase II trials published during a 5-year period in 3 journals in oncology, following the Key Methodological Score. The secondary endpoint was to identify factors associated with a high value of the reporting score.

## Methods

### Study selection

Three oncology journals were randomly selected among a selection of oncology journals with a 5-year Impact Factor above 4. Journals retained were Journal of Clinical Oncology, Annals of Oncology and British Journal of Cancer. All phase II and phase II/III trials published during a 5-year period, between March 2010 and February 2015, in these 3 journals, were eligible. Phase I/II trials, reanalysis of the subgroup of patients included in phase II or II/III trials, pooled analysis of many phase II or II/III trials, publications reporting only the phase III part of phase II/III trials, were excluded. Phase II or II/III trials with the Bayesian design were also excluded. The choice of excluding phase II and II/III trials with the Bayesian design is justified by the difference in the methodology of these trials, compared to the non-Bayesian phase II or II/III trials. We assume that analysis of reporting in these types of trials cannot be realized with the same score as the non-Bayesian trials and that inclusion of these trials would have led to heterogeneity in the set of analyses.

The selections of studies were independently realized by two of the authors and then compared. One of the authors manually selected each phase II and phase II/III trial published during the concerned period in the journal’s computerized archives, with the following key-words: “phase II” or “phase 2” or “phase II/III” or “phase 2/3” present in the title and/or in the abstract and/or in the last paragraph of the Introduction and/or in the Methods section of the article. The other author selected studies with the following electronic request in the MEDLINE database: ((("Br J Cancer."[Journal] OR "J Clin Oncol."[Journal]) OR "Ann Oncol."[Journal]) AND ("2010/03"[PDAT]: "2015/02"[PDAT])) AND Clinical Trial, Phase II[ptyp]). All the publications identified with the electronic request were then manually selected by this author in order to keep only phase II and phase II/III trials that were published in the 3 defined journals. Selected trials by both authors were conserved for analysis. Discordant articles between the two selection methods were read by a third author in order to make the final decision as to whether to include the publication for analysis. The present systematic review was not registered in PROSPERO.

### Study analysis

A data extraction sheet was developed by a multidisciplinary team composed of a methodologist, a statistician and clinicians. It was then pilot-tested on ten randomly-selected included studies, and it was refined accordingly. One author extracted the data from included studies. The following variables were collected: year of publication, disease site, type of treatment, drug combination or not, type of chemotherapy in case of a chemotherapy trial, biomarker analysis (yes/no), statistical design used, reporting of the statistical design (yes/no), comparative trial or not if more than one arm, type of statistical plan, number of stages, random ratio when randomized trial, type I and type II error values, a priori hypothesis (yes/no), cut-off point to conclude for non-comparative trials (present/missing), percentage of loss to follow-up planned, type of funding, planned intermediate analysis (yes/no), early stopping on planned intermediate analysis or not, number of centers, trial results, type of primary endpoint, number of patients anticipated, included and analyzed, type of analysis planned and realized. Trials for which the author had difficulty in completing the data collection base, were read by a second author to make a decision.

### The Key Methodological Score items collection

The use of the Key Methodological Score to analyze the reporting quality of oncology phase II trials have previously been reported [27]. The three following items were systematically recorded for each publication: reporting of the primary endpoint, reporting of the elements needed to justify the sample size calculation, reporting of the definition of an evaluable population (Table 1). The primary endpoint was considered to have been reported when described in the Introduction or in the Methods section of the article. When primary endpoint was reported only in the abstract of the publication, it was considered not to have been reported. Justification of sample size calculation needed the reporting of at least the a priori hypothesis, as well as the type I and type II error values. The definition of the evaluable population was screened and defined as a similar population selection and assessment, planned in the Methods section and realized in the trial analysis. One point was attributed for each item if reported in the publication. Minimal and maximal scores could be respectively 0 and 3.

**Table 1:** Key Methodological Score as published by Grellety et al. [27].

Items	Definition
Primary end point	Clear referencing and definition of the first end point
Sample size	Hypothesis and justification of sample size clearly explained
Evaluable population	Definition of rules to consider patients as being evaluable for first and secondary end points

### Statistical analysis

Descriptive analysis was performed: qualitative variables were described using frequencies and percentages. The Key Methodological Score was described with rate (score of 0, 1, 2 or 3) and median score (interquartile range and minimal and maximal values). Secondary, univariate and multivariate analyses were performed in order to explore variables potentially associated with the highest score of the Key Methodological Score (equal to 3) versus all other scores (lower than 3). The following variables were included in the univariate analysis: journal of publication, year of publication, site of disease, statistical design used, reporting of the statistical design, drug combination, non-comparative versus comparative analysis, type of random ratio, biomarker analysis, reporting of the cut-off point to conclude, type of funding, early stopping on intermediate analysis, number of centers, results of trial, type of primary endpoint, anticipated number of patients to include, comparison between number of patients anticipated/included and anticipated/analyzed, type of analysis planned and realized. A logistic regression was conducted in order to obtain an odds ratio for each variable, with a 95% confidence interval (CI). Variables identified with a p-value lower or equal than 0.2 in univariate analysis and showing no correlation, were included in a multivariate model and analyzed with a logistic regression. Variables with a p-value lower than 0.05 after multivariate analysis were considered as significantly associated with the the Key Methodological Score value. All the statistical analyses were conducted with R 3.1.1 software.

## Results

### Study selection

**Fig 1:**
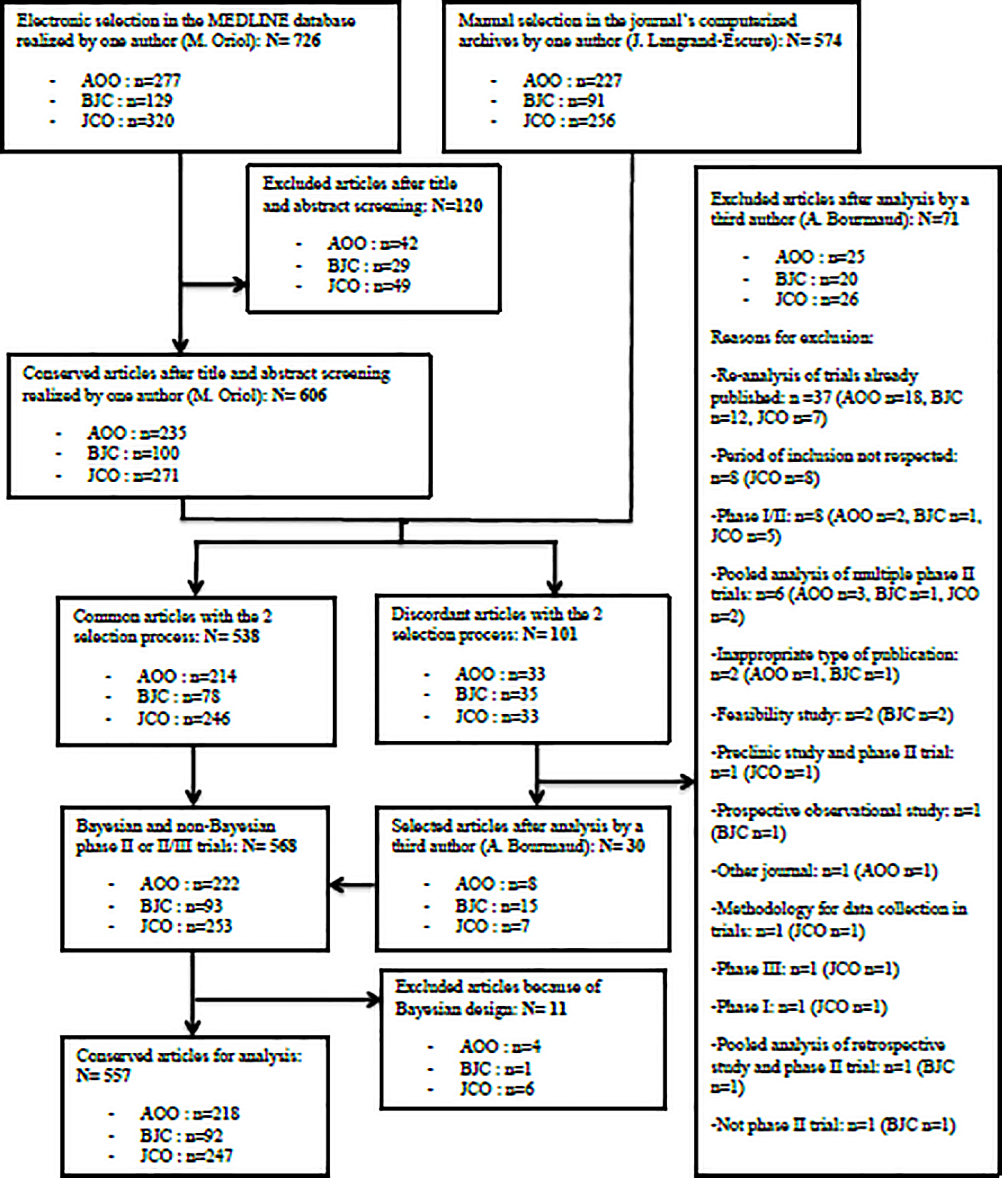
Flowchart. —Flowchart summarizing the selection process of the phase II trials for analysis (AOO: *Annals of Oncology*; BJC: *British Journal of Cancer*; JCO: *Journal of Clinical Oncology)*.

Authors identified 574 and 606 phase II or II/III trials of interest, respectively through manual selection in the journal’s computerized archives and by electronic request in the MEDLINE database plus manual screening. The 2 selection processes identified 538 publications in common. The analysis of discordant articles realized by a third author lead to the conservation of 30 more publications (568 publications). After exclusion of the Bayesian design trials, 557 phase II or II/III trials were retained for analysis with respectively 247 (44.4%), 218 (39.1%) and 92 (16.5%) articles in *Journal of Clinical Oncology*, *Annals of Oncology* and *British Journal of Cancer*. The whole study process selection is described in the flow-chart (Fig 1).

### Study characteristics

Selected trials were essentially single-arm studies (n = 315, 56.6%). 193 (34.6%) trials were randomized and 49 (8.8%) were multiple-arm, non randomized trials. Study design was clearly reported for 395 trials (70.9%), whereas it was deduced but not clearly announced for 162 publications (29.1%). Most of the trials that were analyzed were testing chemotherapy (78.3%). The three sites of disease with which we were most concerned, were gastrointestinal tract (22.3%), hematological disease (15.3%) and lung cancer (12.6%). 443 (79.5%) trials were multicenter trials, whereas 43 (7.7%) were single-center trials and for 71 (12.7%) trials this data was not reported. All study characteristics are detailed in Table 2.

**Table 2:** Characteristics of phase II and II/III trials published in the 3 journals selected (N = 557).

Characteristics		Number of articles (%)
**Journal title**	Journal of Clinical Oncology	247 (44.4)
	Annals of Oncology	218 (39.1)
	British Journal of Cancer	92 (16.5)
**Year of publication**	2010	107 (19.2)
	2011	120 (21.5)
	2012	107 (19.2)
	2013	125 (22.4)
	2014	81 (14.5)
	2015	17 (3.1)
**Type of treatment**	Intravenous chemotherapy	319 (57.3)
	Non intravenous chemotherapy	117 (21)
	Radiochemotherapy	51 (9.2)
	Hormonal therapy	11 (2)
	Immunotherapy	11 (2)
	Radiation therapy	6 (1.1)
	Other	42 (7.5)
Drug combination (*)	Yes	312 (62.4)
	No	188 (37.6)
Type of chemotherapy (*)	Combination	193 (39.5)
	Cytotoxic	148 (30.3)
	Targeted therapy	103 (21.1)
	Monoclonal antibody	36 (7.4)
	Non assessable	9 (1.8)
**Type of neoplasm**	Gastrointestinal tract	124 (22.3)
	Hematology	85 (15.3)
	Lung	70 (12.6)
	Breast	69 (12.4)
	Urology	51 (9.2)
	Gynecology	38 (6.8)
	Head and neck	32 (5.7)
	Skin	23 (4.1)
	Sarcoma	17 (3.1)
	Many sites	12 (2.2)
	Brain	11 (2)
	Other	25 (4.5)
**Type of study**	Phase II study	554 (99.5)
	Phase II/III study	3 (0.3)
**Study design**	Single arm	315 (56.6)
	Randomized trial	193 (34.6)
	Multiple arm non randomized trial	49 (8.8)
**Reporting of the study design**	Yes	395 (70.9)
	No	162 (29.1)
Randomized trial (*)	Comparative	117 (60.6)
	Non-comparative	76 (39.4)
Multiple arm non randomized trial (*)	Comparative	6 (12.2)
	Non-comparative	43 (87.8)
Random ratio (*)	Well-balanced	140 (72.5)
	Non-well-balanced	19 (9.8)
	Non assessable	34 (17.6)
**Type I error value**	<5%	28 (5)
	5%	210 (37.7)
	]5–10%]	132 (23.7)
	>10%	26 (4.7)
	Non assessable	161 (28.9)
**Power value**	<80%	15 (2.7)
	80%	157 (28.2)
	]80–90%[	50 (9)
	90%	141 (25.3)
	>90%	33 (5.9)
	Non assessable	161 (28.9)
**A priori hypothesis**	Present	461 (82.8)
	Missing	96 (17.2)
**Analysis of biomarker**	Yes	291 (52.2)
	No	266 (47.8)
**Study funding**	Private	322 (57.8)
	Private and public	76 (13.6)
	Public	58 (10.4)
	No funding	36 (6.5)
	Not reported	65 (11.7)
**Number of center**	Multicenter	443 (79.5)
	Single center	43 (7.7)
	Not reported	71 (12.7)
**Study results**	Positive	245 (44)
	Negative	152 (27.3)
	Non assessable	160 (28.7)
Cut-off point to conclude (*)	Present	246 (56.7)
	Missing	187 (43.1)
	Non assessable	1 (0.2)
Type of primary end point (*)	Rate	372 (80.7)
	Censored data	62 (13.4)
	Incidence	6 (1.3)
	Other	21 (4.6)
Number of primary end point (*)	Unique	461 (91.5)
	Multiple	43 (8.5)
**Type of analysis planned**	Not reported	287 (51.5)
	Intent to treat	152 (27.3)
	Per-protocol	116 (20.8)
	Other	2 (0.4)
**Type of analysis realized**	Per-protocol	335 (60.1)
	Intent to treat	215 (38.6)
	Other	7 (1.3)

(*) subgroup descriptive analysis

### Key Methodological score

The median value of the Key Methodological Score was 2 (interquartile range: 1–3). Key Methodological Score was equal to 0, 1, 2, 3 respectively for 22 (3.9%), 119 (21.4%), 270 (48.5%) and 146 (26.2%) trials. Detailed scores for each item of the Key Methodological Score are described in Table 3. Concerning univariate analysis, disease site, type of statistical design, reporting of statistical design, cut-off point to conclude, number of center, trial results, anticipated number of patients to include, comparison between number of patients anticipated/included and anticipated/analyzed, as well as type of analysis planned and realized, were significantly associated with the highest KMS value. Concerning multivariate analysis, 3 variables were significantly associated with the Key Methodological Score value (equal to 3 versus <3): reporting of statistical design (OR = 2.22; IC 95% [1.36–3.65]; p-value <0.001), single center trial versus multicenter trial (OR = 0.25 [0.09–0.74]; p-value = 0.012) and analysis conducted in intention to treat versus per-protocol (OR = 0.48; IC 95% [0.32–0.72]; p-value <0.001). The results of the multivariate analysis are summarized in Table 4.

**Table 3:** Details of rate of reporting of each item of Key Methodological Score.

Items	Number of trials with item reported (%)
Primary endpoint	504 (90.5)
Sample size justification	371 (66.6)
Evaluable population’s definition	222 (39.9)

**Table 4:** Factors associated with Key Methodological Score value: Multivariate analysis results.

Variables		Odds ratio ; 95% CI	p-value
**Reporting of statistical design** (reported versus deduced)		2.22 [1.36–3.65]	**<0.001**
**Number of center** (reference: multicenter)			**<0.001**
	Single center	0.25 [0.09–0.74]	0.012
	Non assessable data	0.39 [0.19–0.79]	0.009
**Type of analysis conducted** (reference: intent-to-treat)			**<0.001**
	Per-protocol analysis	0.48 [032–0.72]	<0.001
	Non assessable data	0 [0-∞]	0.981

## Discussion

The quality of reporting in oncology phase II and II/III trials can be improved according to the low number of clinical trials (26.2%) that reported all three items of the Key Methodological Score in our set of analysis. The reporting of statistical design, multicenter trial and trial analysis conducted in intent-to-treat are significantly associated with the highest value of the Key Methodological Score (equal to 3) in this analysis of 557 oncology phase II and II/III trials. On the other hand, no significant association was found between the Key Methodological Score and journal of publication, year of publication and type of statistical design. A further analysis of this review brought to light several issues. First, the three items of the Key Methodological Score are not reported in the same proportions: the primary end point is almost systematically reported, while sample size calculation is missing in one third of the articles. The definition of population is the least reported item, with more studies failing to report such information, than studies reporting it. The definition of the population being one of the 3 core components of the Key Methodological Score, this very low rate may raise questions concerning the validity of such studies. The second issue results from the other items collected in the extraction sheet as descriptive characteristics: more than half of the studies are single-armed, one third of the studies have failed to report the study design which could be considered as a major methodological requirement, 20% are not cited as being multicenter, 20% do not provide a research hypothesis, 11% do not provide their funding sources and 43% do not report their cut off point. All those reporting items are considered as mandatory in other study design reporting guidelines. The extent of the defect in such items also raises questions concerning the validity of these studies.

To our knowledge, only 2 publications assessed reporting in oncology phase II trials with a global score [27,28]. Ottaiano *et al* [28], published an 11-item score, called the Quality Index which is adapted to phase II trials of biotherapy and immunotherapy in oncology. 141 trials published in 5 oncology journals, during a 5-year period, between 1998 and 2002, were analyzed. The Impact Factor of the journal was significantly associated with a score higher than 50/100 (p-value = 0.001). Contrary to Ottaiano *et al*, our analysis with 3 oncology journals found no association with the journal type and reputation, even if the 5-year Impact Factor of the 3 journals selected was well distributed: 16.97 for Journal of Clinical Oncology, 6.89 for Annals of Oncology and 5.31 for British Journal of Cancer. Furthermore, the distribution of trials selected in our analysis were well balanced with 247 trials selected in Journal of Clinical Oncology (Impact Factor >10) and 310 trials selected in Annals of Oncology and British Journal of Cancer (Impact Factor <10).

Grellety *et al* [27] published two global scores for the assessment of reporting quality for oncology phase II trials: the Overall Quality Score and the Key Methodological Score. The first one could be considered, like the CONSORT guidelines, as an aid for the writing of trials, for authors considering the 44 items proposed. The Key Methodological Score, which includes only 3 items, is in fact, from our point of view, the only tool adapted to systematic reporting evaluation in oncology phase II trials. These scores were used by the authors for the analysis of anticancer drugs, radiotherapy and surgery trials. They screened 156 trials published in 8 oncology journals with a 5-year Impact Factor above 4. The period of selection was the year 2011. Rates of reporting of each item of the Key Methodological Score was 68.6%, 77.6% and 33.3% respectively for the reporting of primary endpoint, reporting of the sample size justification and reporting of the definition of an evaluable population. These rates seem to be comparable with our findings for the two last cited items: 66.6% versus 77.6% for sample size justification and 39.9% versus 33.3% for the definition of an evaluable population. Concerning the reporting of primary endpoint, we found a rate higher than Grellety *et al* (90.5% versus 68.6%). Whereas these authors’ analysis concerned a larger pool of oncology journals than ours, our analysis concerned a 5-year period which could explain the higher number of screened trials (557 versus 156). A statistical analysis was also conducted by Grellety *et al*. No factor was associated with Key Methodological Score value, on both univariate and multivariate analysis. However, variables explored by the authors on univariate and multivariate analysis were not as numerous as our variables. Research funding, site of disease and type of treatment were the only similar variables analyzed in the two studies. Impact Factor higher than 10 and registration on clinicaltrials.gov were significantly associated in multivariate analysis to improve the Overall Quality Score.

The quality of reporting was also analyzed on a unique criteria. Reporting of the statistical design appeared to be the most frequently studied criteria [17–23]. Like us, Perrone *et al* [20], showed a significant impact of the number of participating centers on reporting quality, in 145 breast cancer phase II trials, published between 1995 and 1999. Multicenter trials were significantly associated with a higher rate of statistical design reporting (OR = 3.24; 95%CI [1.47–7.15]). In this study, single drug trial, journal Impact Factor and the period of time between the beginning of the study and its publication, were also significantly associated with statistical design reporting.

Some authors analyzed the rate of reporting of one of the three items of the Key Methodological Score. Sample size justification was the object of two descriptive analyses, but no statistical analysis was conducted in order to find factors associated with this criteria [23,29]. Reporting of the primary endpoint in oncology phase II trials was analyzed descriptively [23,30]. Nickolich *et al* [31] showed that the rate of primary endpoint reporting increased significantly with time in 40 small cell, lung cancer phase II trials published in the *Journal of Clinical Oncology* between 1986 and 2010. Indeed, primary endpoint was reported in 10%, 55.6%, 100% respectively for the periods 1986–1996, 1997–2005, 2006–2010 (p-value = 0.01). No significant association was found in our analysis between the year of publication and the Key Methodological Score. We note that the Key Methodological Score publication by Grellety *et al* [27] in January 2014 did not improve the reporting quality of oncology phase II trials during the period January 2014-February 2015 in our analysis. This could be explained by the very short period of time between its publication and the end of our extraction: these new recommendations had yet to be adopted by the scientific community.

One of the biases of our analysis is that phase II trial screenings were performed by mainly one author. Double reading of phase II trials was done only for the studies for which the reviewer hesitated to fill in the data base. Inversely, Ottaiano *et al* [28], and Grellety *et al* [27] assessed each trial with at least two reviewers.

Furthermore, inclusion in the analysis of phase II/III trials can be questionable. Indeed, in Grellety *et al* publication [27], phase II/III trials were excluded from analysis. However, we analyzed only phase II part of phase II/III trials, and we undertake that there is no difference in terms of the need for the quality of reporting between phase II alone and phase II part of phase II/III trials.

The Key Methodological Score, used as a reference score to evaluate the quality of reporting of oncology phase II trials, is debatable. Indeed, with only 3 items, the boundary between “good” and “bad” reporting is difficult to determine. Studies with a score equal to 2 are the most frequent in our analysis (48.5% of trials). On the other hand, the 3 items of the Key Methodological Score, present advantages of reproducibility and facility of use for the reader. The choice of using this score for our analysis was justified by the fact that it was, to our knowledge, the only global score published for systematic analysis of reporting quality for oncology phase II clinical trials. Yet, improvement and refinement of the Key Methodological Score would be of interest, in order to get closer, in its structure, to the other widely spread methodological reporting assessment scores.

This study supports the assumption of non-optimal reporting for the phase II clinical trials in scientific published articles. It also raised the question of the design quality and of the suitability of the methodological choices in the conduction of these trials. This issue deserves further investigation. Our analysis enriches the poor content in the scientific literature on the subject of the reporting quality of phase II oncology trials, which are a cornerstone of the development of new anticancer therapies. This is to our knowledge, the largest study in terms of the quantity of analyzed trials, and the first to demonstrate a statistically significant association between some study characteristics and the Key Methodological Score value. Because no other global score is available today, the Key Methodological Score must be considered as a reference for the systematic analysis of the quality of reporting in phase II oncology trials, both by authors and readers.

## Supporting information

S1 Table Data set supporting the analyses. (XLSX)

S2 Table PRISMA statement check list. Authors are designed by their initials.
(DOC)
